# Applied Graph-Mining Algorithms to Study Biomolecular Interaction Networks

**DOI:** 10.1155/2014/439476

**Published:** 2014-04-02

**Authors:** Ru Shen, Chittibabu Guda

**Affiliations:** ^1^Department of Computer Science, University at Albany, 1400 Washington Avenue, Albany, NY 12222, USA; ^2^Department of Genetics, Cell Biology and Anatomy, University of Nebraska Medical Center, College of Medicine, Omaha, NE 68198-5145, USA; ^3^Bioinformatics and Systems Biology Core, University of Nebraska Medical Center, Omaha, NE 68198, USA

## Abstract

Protein-protein interaction (PPI) networks carry vital information on the organization of molecular interactions in cellular systems. The identification of functionally relevant modules in PPI networks is one of the most important applications of biological network analysis. Computational analysis is becoming an indispensable tool to understand large-scale biomolecular interaction networks. Several types of computational methods have been developed and employed for the analysis of PPI networks. Of these computational methods, graph comparison and module detection are the two most commonly used strategies. This review summarizes current literature on graph kernel and graph alignment methods for graph comparison strategies, as well as module detection approaches including seed-and-extend, hierarchical clustering, optimization-based, probabilistic, and frequent subgraph methods. Herein, we provide a comprehensive review of the major algorithms employed under each theme, including our recently published frequent subgraph method, for detecting functional modules commonly shared across multiple cancer PPI networks.

## 1. Introduction

Recent advances in systems biology research have generated a wealth of data on physical and genetic interactions capable of revealing relationships between biomolecules. For example, high-throughput screening methods, such as two-hybrid analysis [1] and mass spectrometry [2], have produced volumes of data on protein-protein interactions (PPI). PPI networks provide the basis for understanding the modular organization of molecular interactions. Still, computational algorithms are required to process this data for large-scale PPI networks. Such networks provide the basis for understanding the modular organization of molecular interactions. Analyzing PPI networks using graph-theory-based algorithms and graph-mining methods has become commonplace in systems biology research. Similarly, for comparative analysis of PPI networks in cancer, both graph comparison and module detection have been used to determine the structure of networks [3]. Here, we review various graph comparison and module detection algorithms that have been widely used for analyzing PPI networks in different systems biology applications.

The first group of algorithms of interest, graph comparison, is the process of comparing and contrasting graph-based networks in order to determine the PPI network similarities or detect common or distinct substructures (i.e., subnetworks or subgraphs). Thus, graph-theory-based methods are widely used in the comparative study of the molecular interaction networks (MINs). These methods have been applied in various studies and analyzed in previous review articles. For example, in 2006, Sharan and colleagues published a review of the applications of graph comparison methods to analyze MINs [4]. In our paper, we focus on more current algorithmic details of graph comparison methods. Our primary focus is on the following two most widely published graph comparison algorithms: graph kernels and graph alignments.

The second group of algorithms, module detection, involves the identification of functionally important substructures within a larger PPI network, which is one of the most widely studied topics in PPI network analyses. Considering that biological interactions do not operate based on sequence homology between partners, sequence homology-based methods such as Basic Local Alignment Search Tool (BLAST) [5] are generally not useful for detecting interacting modules. Instead, network analysis has become a key approach to understanding the functional relationships between interacting proteins. Because subunits of a molecular complex generally function towards a common biological goal, predicting an uncharacterized protein as part of a known complex increases the confidence in the annotation of that protein [6]. Thus, detecting important modules in PPI networks is a well-studied problem in graph analysis. In this paper, we review the following five categories of module detection methods: (1) seed-and-extend, (2) hierarchical clustering, (3) optimization-based, (4) probabilistic, and (5) frequent subgraph.

Graph comparison can be used to search conserved regions representing functional, orthologous modules across different species or biological systems. In contrast, module detection algorithms can be applied to graph alignment to find the optimal local alignment between protein networks [7]. Sometimes, both types of algorithms are also used in combination to perform PPI network analysis. For example, finding the common PPI network modules in multiple cancer networks requires the combination of graph comparison and module detection algorithms [3]. Hence, graph comparison and module detection are interrelated rather than isolated topics. This paper is organized as follows. First, we review graph comparison strategies that include graph kernel and graph alignment methods. Next, we discuss module detection strategies that include five subsections covering seed-and-extend, hierarchical clustering, optimization-based, probabilistic, and frequent subgraph methods. The final section includes a summary of this review along with our conclusions.

## 2. Graph Comparison Strategies

Graph comparison is an important tool for understanding PPI networks. For instance, by measuring the discrepancy between PPI networks of healthy and diseased individuals it is possible to predict disease outbreak and progression [8]. Also, through the alignment of networks we can identify evolutionarily conserved patterns in biological pathways [9]. The key to accurate graph comparison is to find a suitable scoring function that can correctly measure differences between networks. Some of the early distance-based methods, including the Maximal Common Subgraph (MCS) [10, 11] and edit distance-based [12] methods, focus on the global comparison of networks. For the MCS method, similarity is measured based on the percentage of the overall network that the maximal common subgraph occupies. For the graph edit distance method, similarity is measured by what it takes to transform one network into another by substituting, deleting, and inserting nodes and edges. While distance-based methods provide an intuitive means of comparing graphs, these comparisons are based on the exact matching of substructures and therefore cannot be generalized to identify approximate similarities required for the analyses of many biological networks.

Graphlets are smaller units of subgraphs of distinct sizes. Graphlet degree distribution has been established as a more-comprehensive model compared to early graph comparison methods. Graphlet degree distribution is a generalization of degree distribution of larger networks. In 2006, Przulj reported that agreement in graphlet degree distribution could be effectively used to compare biological networks [13]. This distribution is calculated as the number of nodes attached to each of a predefined number of graphlets. Due to the topological differences of nodes on graphlets, connections at unequal positions of graphlets are considered different attachments. For example, for the 30 predefined graphlets in Przulj's study, there are 73 types of attachments (also known as orbits), which give rise to 73 distributions, one for each orbit. Given two networks for a particular orbit, the distribution agreement of the orbit is the inverse of the Euclidean distance between the two distributions. The distribution agreement between two networks is the arithmetic or geometric mean of the distribution agreement of all orbits. The graphlet degree distribution method was the first of a group of graphlet-based methods. The concepts of graphlet degree and the measurement of similarity by graphlet degree vectors provide the foundation for the subsequent graphlet-based comparison methods, including GRAph ALigner (GRAAL) [14] and its variations, H-GRAAL [15] and MI-GRAAL [16], discussed later in this review.

Like the graphlet degree distribution method that uses graphlets to compare PPI networks, graph kernels decompose networks into subunits and use the subunit information to calculate PPI network similarities.

### 2.1. Graph Kernels

As proposed by Haussler, graph kernels can be viewed as special cases of R-convolution kernels [17]. Graph kernels can offer an analysis of networks by comparing nontrivial substructures. A fundamental issue in graph comparison is the problem of subgraph isomorphism. In this case, given two graphs, G and H, for example, we need to determine whether G contains a subgraph that is isomorphic to H. Subgraph isomorphism is proven to be NP-complete, which presents a large challenge with respect to computational complexity and run time. In the context of graph kernels, in order to accurately compare two PPI networks, an exhaustive comparison of all of their subgraphs is needed. However, performing such a comparison using graph kernels requires the same computational complexity as subgraph isomorphism. Therefore, a key reason for using graph kernels is to closely approximate an exhaustive comparison while maintaining computational tractability. Among various graph kernels,* walk-based kernels* [18, 19] compare graphs by counting the number of matching walks within two input graphs. A walk is a sequence of edges from the graph that connect to a sequence of vertices. Vertices are allowed to repeat in a walk but not in a path.* Path-based kernels* [20] use paths instead of walks to avoid “tottering” problems related to walk-based kernels (i.e., high similarity values resulting from cycles or loops in the graphs). On the* other hand*, subtree kernels compare all pairs of matching substructures in subtree patterns [21, 22]. While subtrees are more expressive structures compared to paths or walks, constructing subtrees is computationally expensive. Here we will highlight a few methods that use fast algorithms for efficient computation of kernels.

In 2005 and 2007, Borgwardt and colleagues proposed fast algorithms for computing random walk kernels [8, 23]. This type of computation performs random walks on graphs in order to decompose these graphs into multiple paths and compute the number of matching paths. Various incarnations of these kernels use different metrics for computing similarities between paths [18, 23], but performance issues when computing large networks plague almost all of these kernels. As a noteworthy advancement, Borgwardt and colleagues introduced the fast random walk kernel that increases the speed of performance to up to three orders of magnitude. In Borgwardt's method, the kernel is defined by the product graph, which is a composite graph whose nodes are tuples of vertices from each network. Edges exist only if the corresponding vertices are adjacent in both networks. Three efficient schemes are utilized to decrease the computation time. These schemes include reducing the kernel to the problem of solving a generalized Sylvester equation [24], using the conjugate gradient methods to solve the kernel and implementing a fixed-point iteration method to speed up the computation time [25].

Given that decomposing networks to small substructures is an expensive process, many state-of-the-art graph kernels do not scale to large graphs. To address this issue, in 2009 Shervashidze and colleagues proposed a statistical approach to compare graphs based on the distribution of graphlets [26]. Here, the graphlet kernel is calculated as the product of the graphlet distribution vectors. Graphlet distribution could be used to represent the distribution of the graph, especially when the graph is large. Given that the exhaustive enumeration of graphlets is prohibitively expensive, two theoretically grounded alternatives have been proposed. First, sampling a fixed number of graphlets suffices to bind the deviation of the empirical estimates of the graphlet distribution from the true distribution. Second, for graphs of a bounded degree, the exact number of all graphlets of size *k* can be determined at a computational complexity that is close to polynomial time. Because the sampling technique of graphlet kernels is independent of the graph size, graphlet kernels are scalable to larger graphs.

In 2007, Shervashidze and colleagues proposed another algorithm for fast computation of subtree kernels [27]. The fast subtree algorithm is built upon the Weisfeiler-Lehman test of isomorphism [28]. The key idea of this algorithm is to first assign the nodes with a sorted set of labels from neighboring nodes and then compress this sorted set of labels to short labels. Given the limited range from elements of the set, this method applies a counting sort on the set of labels to achieve linear complexity. Not only is the runtime reduced, but also the accuracy of fast subtree kernel is competitive with state-of-the-art kernels on several graph classification benchmark data sets. Following the fast subtree kernel, a family of Weisfeiler-Lehman kernels was later designed [29], including the Weisfeiler-Lehman edge kernel and the Weisfeiler-Lehman shortest path kernel. In terms of runtime on large graphs, these kernels outperform other kernels, including the recently developed random walk kernels [8] and graphlet kernels [26].

Graph kernels bridge the gap between graph-structured data and a large spectrum of machine-learning algorithms [29]. Graph kernels have quickly developed into an independent branch of graph-mining methods and are widely used in various areas such as computational biology and social network analysis. Nevertheless, with a single value being produced as a result of the comparison, graph kernels cannot provide detailed information on the node or edge mapping. On the other hand, graph alignment methods are designed to address the above limitations in graph kernel methods. Graph alignment methods can provide a more in-depth knowledge of the comparison, with the tradeoff being a longer processing time.

### 2.2. Graph Alignment Methods

Graph alignment is the process of mapping nodes and edges between graphs such that conserved subgraphs can be identified. Graph alignment adopts a similar concept as that used for sequence alignment. However, in contrast to sequence alignment, which aligns linear sequences to identify regions of similarity, graph alignment must be able to handle data from multiple dimensions of the graph. Graph alignment involves a subgraph isomorphism test that is proven to be NP-complete. Similar to graph kernels, getting an exact solution for graph alignment is not feasible for even moderate sized graphs. Thus, most graph alignment methods resort to heuristic solutions to reduce the cost of computation.

Similar to sequence alignment strategies, graph alignment can be local or global. Local graph alignment matches nodes and edges to maximize the local alignment score. In 2006, Koyuturk and colleagues proposed a local alignment framework for PPI networks based on the duplication/divergence evolutionary model [7]. In this framework, in a given pair of PPI networks, the alignment score between two PPI networks is calculated based on matches, mismatches, and duplications. This method is heuristic-based and aims to locate all maximal protein-subset pairs such that the alignment score is locally maximized. Another example of local alignment is the modular subgraph alignment algorithm [30], where each larger network is decomposed into a collection of smaller subnetworks in order to compute the alignment of the two networks as the optimal alignment of the subnetworks.

A series of NetworkBLAST algorithms have been reported for the global alignment of networks. For example, PathBLAST [9] is the first one of such algorithms to search for high-probability pathway alignments between two PPI networks [31]. A later version, NetworkBLAST [32, 33], constructs a general framework for comparing more than two protein networks in order to search for conserved patterns such as short linear pathways and dense clusters of complexes. This search algorithm exhaustively identifies high scoring subnetwork seeds and uses them to expand the search. NetworkBLAST is an exhaustive approach and is limited in its application to the alignment of only up to three networks, while an extended version, NetworkBLAST-M, can handle multiple networks [34]. NetworkBLAST-M progressively constructs a layered alignment graph, with each layer corresponding to a network. Connections between layers indicate similar proteins across different protein networks. The set of potentially orthologous proteins is represented by a subnet, which includes a vertex from each of the layers. NetworkBLAST-M computes a local alignment by readily finding subnets of high, local conservation based on inferred phylogeny. With the novel representation of a layered alignment graph, NetworkBLAST-M can achieve dramatic reductions in run time and memory requirements for multiple network alignments.

Other methods use biological features of interacting proteins for graph alignment. For example, using integer quadratic programming, Li and colleagues proposed a PPI alignment algorithm in 2006 based on similarities in both the protein sequence and network architecture [35]. In this method, the alignment of PPI networks is formulated as a combination of the sequence similarity score of proteins and the matching score of protein interactions. A coefficient is introduced to balance the weight between the node and edge similarity of the networks. Integer quadratic programming is used to maximize the alignment score among all feasible combinations of matching scores. Nodes without alignment gaps are selected to construct a minimally connected subgraph within each network; these subgraphs are regarded as conserved patterns.

The GHOST alignment method [36] developed by Patro and Kingsford uses the spectrum of the graph adjacency matrix to measure topological similarities between networks. GHOST performs a global network alignment using a two-phased approach. The first phase employs a seed-and-extend strategy to align high scoring node pairs with their neighbors, which is similar to the way that BLAST functions [5]. The second phase uses a local search method to realign nodes in order to achieve better topological or biological quality. NETAL is another global graph alignment method that was developed by Neyshabur and colleagues [37]. NETAL constructs an alignment score matrix and uses a broad search to find the best alignment between networks. The alignment score matrix is constructed from the similarity and interaction score matrices. The similarity score matrix indicates both topological and biological similarities between nodes; the interaction score matrix represents the approximated number of conserved interactions incident to the nodes. During the process of a broad search, node pairs with maximum alignment scores are selected and aligned. As a result, the interaction score matrix is updated, which in turn impacts the alignment score matrix. The process continues until all of the nodes of one network are aligned to at least some nodes of the other network.

GRAAL (GRAph ALigner) is a global alignment method based solely on the network topology [14]. For each node in a network, a vector of “graphlet degrees” is used to record the number of each kind of graphlet that the node touches. Signature similarity is computed as the distance between two vectors. Alignment of two networks is completed by matching pairs of nodes originating in different networks based on the similarity of their signatures. Although this algorithm operates based on local alignment, it produces global alignment results. H-GRAAL, which is a variation of GRAAL [15], uses the Hungarian algorithm to solve assignment problems and determine the optimal alignments between networks. Given the cost of aligning two nodes, the Hungarian algorithm locates the assignment of all pairs of nodes that yield a minimized total cost of alignment. Additionally, there are other graphlet-based alignment algorithms including MI-GRAAL [16] and the latest C-GRAAL [38]. Like GRAAL, MI-GRAAL is a seed-and-extend approach. However, unlike GRAAL and H-GRAAL, which are purely based on topological information, MI-GRAAL can integrate any type of similarity measures into the model. From these similarity measures, MI-GRAAL computes the alignment confidence score between pairs of nodes. High scoring pairs are inserted into a priority queue and used as seeds during the alignment. Similar to GRAAL and MI-GRAAL, C-GRAAL also uses a seed-and-extend approach. In contrast to GRAAL, which is based on graphlet degree information, C-GRAAL aligns networks based on common information of neighboring graphlets.

Regardless of if the calculation is for local or global alignments, performing efficient and accurate alignments on multiple networks continues to be challenging. Nevertheless, the Graemlin algorithm was the first algorithm capable of performing scalable, multiple network alignments [39]. Graemlin starts from pairwise network alignments. It uses a seed-and-extend algorithm to first identify clusters of proteins as “seeds,” and then it broadly extends the alignment to yield a maximal increase in alignment score. In aligning multiple networks, Graemlin successively aligns the closest pair of networks obtained from the pairwise alignment phase, resulting in the construction of new networks from the alignments. In practice, Graemlin avoids an exponential run time because the constructed networks have small overlaps. The newer version, Graemlin 2.0 [40], is a global alignment algorithm that can adjust scoring function parameters and perform multiple network alignments.

IsoRank [41] is another multiple network alignment method that aims to correspond nodes and edges of input networks to maximize the global “match” between PPI networks. The maximum match is a combination of the following two factors: (1) the size of the common graph determined by mapping and (2) the aggregate sequence similarity between nodes mapped to one another. The IsoRank algorithm first associates a functional similarity score with each possible match between nodes of two networks. Functional similarity shapes the tradeoff between the twin objectives of topological overlapping and high sequence similarity between mapped nodes. This similarity is resolved through eigenvalue computation. In the second stage, mappings between networks are extracted from the functional similarity scores. To align multiple networks, the above processes are repeated for each pair of networks. An improved version of IsoRank, IsoRankN [42], was developed to perform more efficient and highly accurate global alignments over multiple networks. Through the use of spectral partitioning algorithm, IsoRankN can find dense, clique-like clusters that are considered conserved regions among the networks.

As a summary of graph comparisons, we have reviewed an array of methods from early single-feature, distance-based algorithms to the most current multiple network alignment graphs. Early distance-based algorithms are founded on strict matching of network structures, and thus these algorithms are only suitable for simple network comparisons. Graph kernels compare graphs by decomposing and comparing graph substructures, which are based on well-supported statistical analyses and mathematical derivations. This results in more accurate and meaningful comparisons, particularly for approximate structural similarities. However, with a single value being produced as the comparison result, graph kernels cannot provide substantial internal details for the comparison, such as the node and edge mapping. Therefore, graph kernels are most suitable for solving classification problems for small to medium sized graphs. For large sized graph comparison, graph kernels are at disadvantage, because on one hand the kernel calculations are very time consuming for large networks and on the other hand the calculated values are less informative than those of smaller sized networks. To perform detailed comparisons of networks, graph alignments are preferred. Local alignments align subnetworks to maximize the local alignment score. Global alignments, on the other hand, focus on maximizing the overall alignment score. While local alignments may be ambiguous, global alignments typically produce unique mapping between nodes. In recent years, several multiple network alignment methods have been developed. Compared to pairwise alignments, multiple network alignment methods provide greater proof of conservation of the identified subnetworks. Graph alignments are very effective for network comparison and identification of conserved regions in networks. However, due to the multidimensional nature and complexity of graph data, graph alignment algorithms rely on heuristics to derive the optimal solution. The drawbacks in graph alignments are that different heuristics usually result in very different solutions and there are no standards or benchmarks like those available for sequence alignment.

A summary of different strategies used for graph comparisons is provided in Table 1. Here, the first column lists the name of the method, and the second column specifies the comparison strategy used. The third column annotates the methods with keywords such as local, global, pairwise, or multiple. Local versus global indicates if the method is for local graph alignment or global graph alignment. Pairwise versus multiple indicates if the method is a pairwise graph comparison or a multiple graph comparison. The last column of the table refers to the reference number listed in the reference section of this paper.

## 3. Module Detection Strategies

One of the most important applications of biological network analysis is the identification of functionally relevant modules in PPI networks. Similar to social networks and internet-based networks, PPI networks are conjectured to exhibit a power law degree distribution [43]. Proving that PPI networks follow a power law degree distribution requires more rigorous statistical data analysis than that available today [44]; however, it is clear that the connectivity of the PPI networks is centralized around a small number of hub nodes. Such a connectivity pattern indicates that the subgraphs (modules) centered on these hub nodes are important for accomplishing specific biological functions. These modules may be mapped to biological pathways or physically interacting complexes. A variety of module detection algorithms exist in the literature. Here, we review the following five methods on module detection: (1) seed-and-extend, (2) hierarchical clustering, (3) optimization-based, (4) probabilistic, and (5) frequent subgraph methods. Many of these methods are also discussed in a recent review on community detection in graphs by Fortunato [45]. In our paper, we also discuss our recently published work on frequent subgraph method for detecting common functional modules among multiple PPI networks involved in nine different cancers [3].

### 3.1. Seed-and-Extend Approaches

Seed-and-extend approaches predict functional protein modules based on the density of PPI networks. The functional modules are generally initiated from single nodes deemed as central nodes or “seeds,” and new nodes are added to “extend” the subnetworks. Different algorithms have specific metrics for determining when the subnetworks will reach convergence.

The first seed-and-extend approach we will review is Molecular Complex Detection (MCODE). This method detects densely connected regions in large PPI networks that may represent molecular complexes [6]. MCODE generates weights for all vertices based on their local network density and identifies high weight seed proteins. Seeds are expanded outwards by including vertices in complexes whose weights are above a given threshold. This process continues until no more vertices can be added to the complex. The Speed and Performance in Clustering (SPICi) method [46] is another seed-and-extend algorithm used for clustering large biological networks. SPICi uses a heuristic approach to build clusters from an initial seed-connected pair of vertices (*S*) with the highest weight degrees. During the expansion stage, SPICi searches for a vertex with the maximum value of support amongst all the nonclustered vertices adjacent to *S*. The procedure is repeated until all vertices in the graph are clustered.

Both the MCODE and SPICi methods are purely based on network topology. In contrast, Maraziotis and colleagues presented a new method that discovers functional modules from weighted graphs [47]. These modules are obtained by clustering proteins according to their gene expression profiles and then measuring the distances between clusters. First, the seed proteins are selected from complexes that have at least six members and more than 80% data coverage. The neighbors of the seed protein are sorted in a descending degree of significance, and this subset of nodes is named the kernel. Adjacent nodes are iteratively added to the selected kernel. Still, of all the seed-and-extend methods, SPICi is the most useful to cluster larger networks due to its efficient memory utilization algorithm [46].

### 3.2. Hierarchical Clustering

Hierarchical clustering is another group of clustering algorithms widely used for biological data analysis. Hierarchical clustering methods are often applied to gene expression data to determine coexpressed genes, clusters, and outliers [48]. Hierarchical clustering methods can also be applied to PPI networks to identify potential modules from within the networks. Similar to seed-and-extend methods, hierarchical clustering algorithms assume the unbalanced distribution of nodes and edges in networks. These methods hierarchically group objects based on the distance among the objects.

The Protein Distance Based on Interactions (PRODISTIN) method was developed based on the principle that the greater the two proteins in a network share common interactions, the more likely it is that they are functionally related [49]. Using the number of common and distinctive interacting members of the two proteins in an interaction network, PRODISTIN computes the functional distance using the Czekanovski-Dice distance formula [50], which reflects the symmetrical difference between the two proteins in an interaction network. The distance values are clustered using a variation of the neighbor-joining algorithm [51] to generate a hierarchical tree. In 2004, Lu and colleagues presented ADJW and Hall clustering algorithms [52]. ADJW employs the adjacency matrix of the network as the similarity matrix for the clustering. Densities of the edges between node groups are computed from the similarity matrix. Using single linkage clustering, each step clusters two groups having maximum edge densities into one group. Hall clustering projects proteins into Euclidian space according to their connectivity. The Euclidian distances are used as a metric to measure the topological distance of vertices in the network. Because the two groups with the smallest sum form a new group at each step, the groups closer in distance are selected earlier in the clustering process.

The hierarchical clustering methods discussed above are agglomerative methods in which the additions of edges are used to construct hierarchical trees. In hierarchical clustering, there is another class of methods called divisive methods that construct hierarchical trees by removing edges. Divisive methods attempt to find the least similar connected pairs of vertices from the network of interest and then remove the edges between the pairs. An example of a divisive method is Newman and colleagues' hierarchical clustering for finding community structures in networks [53]. This method looks for edges with the highest “betweenness,” where betweenness is a measure that favors edges that lie between communities and disfavors edges that lie inside communities. By removing the edges with highest betweenness, this method divides the network into smaller components.

Hierarchical clustering methods have primarily focused on grouping nodes. An unconventional method proposed by Ahn and colleagues in 2010 focuses on edge clustering [54]. Rather than assuming that a module is a set of nodes connected to one another, edge clustering defines modules as sets of closely interrelated links. Similarity scores are calculated for each pair of links that share a node; this calculation is used to build a link dendrogram. An objective function is defined to compute the link density at each level of the dendrogram to help determine the best way to cut the tree, so to speak. Compared to node-based clustering, edge-based clustering can simultaneously reveal hierarchical and overlapping relationships. In another work related to edge-based clustering, Solava and colleagues proposed a measure of edge graphlet-degree-vector (GDV) similarity [55]. Edge GDV counts the number of different graphlets that touch an edge. Because their extended network neighbors are compared, the edge GDV of two edges gives a sensitive measure of their topological similarity. When edge GDV similarity is used to substitute the original edge similarity measure, as is the case in Ahn and colleagues' hierarchical clustering method [54], the predication accuracy outperforms the original algorithm.

### 3.3. Optimization Methods

In addition to seed-and-extend approaches and hierarchical clustering, module detection can also be formulated as an optimization problem. In 2004, King and colleagues completed work on predicting protein complexes via cost-based clustering [56]. Here, the module detection problem is transformed to finding the optimum partitioning of the network in order to minimize the value of the clustering cost function. One method, namely, Restricted Neighborhood Search Clustering (RNSC), is a local search algorithm based loosely on the Tabu search metaheuristic [57]. To search for low cost graph partitioning, RNSC uses a simple integer-valued cost function, referred to as the naive cost function, as a preprocessor. Next, a more expressive real-valued cost function, referred to as the scaled cost function, is used to evaluate clustering. RNSC iteratively moves a node from one cluster to another in a randomized fashion to reduce the clustering cost.

Genes with significant changes in expression have immediate and wide interest as markers of disease and the stage of disease development, as well as markers for a variety of other cellular phenotypes [58]. Genes with correlated expression changes in many conditions are likely involved in similar functions or cellular processes. Such correlated expression patterns in networks can be uncovered by subnetwork searching. For example, the Cytoscape plug-in jActiveModules searches for such active subnetworks (i.e., connected regions of the network that show significant changes in expression over particular subsets of conditions) [59]. The jActiveModules method first adopts a statistical scoring system to capture a change in gene expression for a given subnetwork. Then, jActiveModules identifies the highest scoring subnetworks, which are the active modules in the network studied. Because the problem of finding the maximal-scoring-connected subgraph is NP-hard, the heuristic simulated annealing algorithm is used.

In 2010, Zhang and colleagues introduced a new method that uses graph modularity density to detect functional modules in PPI networks [60]. Graph modularity measures the fraction of edges in the network that connect vertices of the same type (community) against the expected value of connections in a random network. The larger the value of modularity density is, the more accurate the partition would be. Therefore, the community detection problem can be viewed as a problem of finding a network partition that has maximal modularity density. The optimization problem of finding maximized modularity density is first attempted using the simulated annealing (SA) technique. SA allows one to complete an exhaustive search of networks and minimize the problem of finding suboptimal partitions. The result of running SA over modularity and modularity density shows that maximizing the density can provide more detailed and valid results.

HOTNET, published by Raphael's lab in 2011, is another framework for de novo identification of significantly mutated subnetworks [61]. HOTNET first formulates an influence measure between pairs of genes based on their topological relationships in the network. Next, HOTNET builds an influence graph that incorporates information from only the mutated genes in the neighboring network. One clear way to detect significant subnetworks from the influence graph is to identify sets of nodes that are connected through a high influence measure and correspond to mutated genes. However, finding such node sets is a difficult problem that cannot be solved in polynomial time by any known algorithms. Alternatively, a computationally efficient approach is based on the concept of enhancing the influence measure by the number of mutations observed in each of these genes. Thus, the strength of connections in the enhanced influence graph is a function of both the interaction between the nodes and the number of mutations observed in their corresponding genes. Next, a threshold is set for the weight of connections, and the influence graph is decomposed into connected components by removing edges with weights smaller than the threshold. The significance of the subnetworks discovered depends on the choice of threshold. The computational complexity of the algorithm is linear to the size of the graph.

### 3.4. Probabilistic Methods

In recent years, probabilistic-based machine learning methods have been developed and successfully used in many areas in bioinformatics. Here, we review a few machine learning methods developed for network module detection. In 2011, Shi and colleagues used a “semisupervised” method for detecting protein complexes in PPI networks [62]. This method uses topological features such as degree statistics, edge weight statistics, clustering coefficients, and biological features like protein length and polarity of amino acids to construct a two-layer, feed-forward neural network. Shi first obtained a weighted PPI network, a set of known protein complexes, and nonprotein complexes to set up the training model. Using the initial model, this method builds new complexes and uses them to train the model iteratively until no more proteins can be added. PPI networks typically contain large amounts of false negative (missing data) and false positive connections. Because neural networks are regarded for their high tolerance for noisy data, this method offers a suitable model for PPI module detection.

In 2008, Qi and colleagues presented a Bayesian network (BN) algorithm for detecting protein complexes from PPI networks [63]. In this supervised learning approach, a probabilistic Bayesian network mimics each complex subgraph. Specific topological and biological features are selected for representative properties of the protein complex. These features include node size, degree statistics, edge weight statistics, and protein weight or size statistics. Another method, the Markov Clustering Method (MCL), is based on the probability of landing on different vertices through random walks in the network [64]. The premises underlying MCL are that (1) the number of paths between two vertices is larger when the two vertices belong to the same cluster and (2) random walks have a higher probability of traversing within the same cluster than traversing across different clusters. The algorithm starts by creating a Markov matrix, which is an adjacency matrix normalized to 1. Two operators, expansion and inflation, are then used iteratively to recompute the transition probabilities. Expansion corresponds to matrix multiplication, which is responsible for creating new edges, while inflation increases the contrasts between existing differences of probability. The iterative process converges quickly, and the resulting matrix represents a nonoverlapping cluster of the network.

### 3.5. Frequent Subgraph Methods

Most module detection algorithms are based on either network connectivity or the density of subgraphs. In contrast, we recently developed a novel method that predicts functional modules based on the frequency of subgraphs [3]. We compared nine cancer PPI networks to identify common and frequent substructures among the networks. Given their unusual frequency in multiple cancer-related PPI networks, these substructures strongly appear to be functionally relevant to cancer. Our method begins with assigning canonical labels to subgraphs, where subgraphs with the same canonical labels are isomorphic to one another. Starting from small sized subgraphs, the canonical labels are compared, and infrequent edges are pruned from the networks. Frequent and common substructures are recorded and included in the search for larger sized modules. The process iterates as the subgraph size increases until no more substructures can be discovered. From the nine cancer PPI networks, frequent and common substructures have been discovered from two to ten edges. Gene Ontology (GO) semantic similarity scores of the substructures discovered have been compared with those of randomly generated patterns [65]. Common substructures exhibit significantly higher scores compared to random substructures at all edge levels, indicating that the discovered subgraphs are functionally significant. A survey of frequency-based subgraph mining algorithms was previously reviewed by Jiang and colleagues [66]. In general, Apriori-based approach and pattern growth approach are the two major groups of algorithms for identifying frequent subgraphs. Apriori-based algorithms such as FSG [67] generate candidate subgraphs of larger size by joining two smaller subgraphs. On the other hand, pattern growth approach [68] extends patterns directly from a single pattern, instead of joining two smaller subgraphs. Nevertheless, both groups of algorithms are restricted by the graph size due to the subgraph isomorphism problem.

In summary, we reviewed multiple methods for module detection. The performance of each of these methods varies. In Brohee and colleagues' review paper, the performance of MCL, RNSC, and MCODE is compared in terms of robustness, sensitivity, and the results of clustering [69]. In general, RNSC and MCL outperform the MCODE under most conditions. Similarly, Dhara and colleagues performed a comparative behavioral analysis of RNSC and MCL on power law distribution graphs [70]. According to their analysis, RNSC is preferred to MCL in terms of both cost and quality.

Among the numerous methods for module detection, different metrics are used to evaluate the weights of modules. Some metrics are based on the connectivity density, such as MCODE and edge clustering, while some are based on vertex scoring, such as RNSC and jActiveModules. Seed-and-extend methods such as MCODE and SPICi assume that hub nodes always exist as the centers of modules. Such assumptions may limit the types of modules that can be discovered by seed-and-extend algorithms. On the other hand, hierarchical clustering algorithms, including both agglomerative and divisive, do not effectively use the topological information of the networks. Because the distances between nodes or edges determine how the clusters are drawn, using different distance metrics in the algorithm will lead to different clustering results. Optimization methods have their limitations too. Each run of optimization methods may generate different results, depending on the initial settings. Therefore multiple runs of optimization methods are required to achieve a relatively consistent result. Finally, frequency-based methods look for recurring patterns in PPI networks. Frequency-based methods are plagued by the performance issue because frequent pattern matching involves subgraph isomorphism tests, and it is proven that subgraph isomorphism problem is NP-complete.

Table 2 provides a summary of these different methods. Similar to Table 1, the first and second columns list the name of the method and module detection strategy, respectively. The third column annotates each method with specifications; topological versus both indicates if the method is purely based on topological information or both topological and biological information. Depending on the theoretical assumptions and metrics included, these methods are capable of uncovering substructures that represent specific biological functions. Deciding which method to use depends on the specific biological context of the problem.

## 4. Conclusions

Graph comparison and module detection are two commonly used strategies for analyzing PPI networks. Among the algorithms for graph comparisons, graph kernels compare graphs by decomposing the graphs to nontrivial subunits. Similarity scores between the graphs can be derived through comparing these subunits. In contrast to other graph comparison methods, graph kernels have the advantage of speed. In the development of graph kernels, efficiency is the key issue addressed. In contrast to graph kernels that can only produce limited information from the comparison, graph alignment provides in-depth analysis of the mappings between graphs. Graph alignment adopts concepts from sequence alignment; the alignment scores are adjusted to reflect topological or relational information for the graphs. For the purpose of graph comparison, graph kernels are suitable for classification tasks that require high-speed computation and intuitive measurement of distance. Graph alignment methods are suitable for determining conserved regions between PPI networks. Note that graph alignments can be local or global, and graph comparisons can be between two networks (pairwise) or greater than two networks (multiple).

Among the module detection algorithms, seed-and-extend methods identify modules by first selecting their core nodes and then expanding the core nodes with new nodes that increase the subgraph density. Hierarchical clustering creates clusters hierarchically based on distances between the clusters. Optimization-based and probabilistic approaches use mathematical derivations to determine best scoring modules. The frequent subgraph approach searches for common and frequent substructures among PPI networks. Different methods tackle the problem from different perspectives. For example, seed-and-extend methods use connection density and neighboring network information to find heavily connected modules in PPI networks. Hierarchical clustering methods use distances between nodes or edges as the key factor for clustering. Optimization-based methods represent network divisions using mathematical models. Modules are detected through the optimal division of the network. Probabilistic methods use statistics of graph data to construct training models and to determine the state transitions of the algorithm. Finally, for frequency-based algorithms, the frequency of subgraphs becomes the key criterion for detecting modules. The method selection for graph analysis depends on the interpretation of the problem and the perspective of the investigator tackling the problem. As the methods are developed from different perspectives, they produce complementary views of graph data. The future development of graph analysis will be likely focused on integrated analysis using an ensemble of methods because no single method can perform well for all types of comparisons. Because most of the interaction network studies require both graph comparison and module detection, such analyses will benefit from the integration of methods.

## Figures and Tables

**Table 1:** A summary of graph comparison methods by the strategy employed.

Methods	Comparison strategy	Specification				References
		Local	Global	Pairwise	Multiple	
MCS	Distance-based		x	x		[10, 11]
Editing distance	Distance-based		x	x		[12]
Graphlet	Graphlet		x	x		[13]
Fast random walk kernel	Graph kernel			x		[8]
Graphlet kernel	Graph kernel			x		[26]
Fast subtree kernel	Graph kernel			x		[27]
Weighted alignment	Graph alignment	X		x		[7]
Substructure-based alignment	Graph alignment	X		x		[30]
Class of NetworkBLAST	Graph alignment	X		x	x	[9, 31–34]
Quadratic programming	Graph alignment	X		x		[35]
Class of GRAAL	Graph alignment		x	x		[14–16, 38]
Class of Graemlin	Graph alignment		x		x	[39, 40]
Class of IsoRank	Graph alignment		x		x	[41, 42]
GHOST	Graph alignment		x	x		[36]
NETAL	Graph alignment		x	x		[37]

**Table 2:** A summary of module detection methods by the strategy employed.

Methods	Module detection strategy	Specification		References
		Topological	Both	
MCODE	Seed-and-extend	x		[6]
SPICi	Seed-and-extend	x		[46]
Kernel set	Seed-and-extend		x	[47]
PRODISTIN	Hierarchical clustering		x	[49]
ADJW and Hall	Hierarchical clustering	x		[52]
Divisive	Hierarchical clustering	x		[53]
Edge clustering	Hierarchical clustering	x		[54, 55]
RNSC	Optimization	x		[56]
jActiveModules	Optimization		x	[59]
Modularity density	Optimization	x		[60]
HOTNET	Optimization		x	[61]
Semi-supervised	Probabilistic		x	[62]
Bayesian network	Probabilistic		x	[63]
MCL	Probabilistic	x		[64]
Frequent subgraph	Frequency-based method	x		[3, 67, 68]
